# Flashing Lights Induce Prolonged Distortions in Visual Cortical Responses and Visual Perception

**DOI:** 10.1523/ENEURO.0304-16.2017

**Published:** 2017-05-12

**Authors:** Genki Minamisawa, Kenta Funayama, Nobuyoshi Matsumoto, Norio Matsuki, Yuji Ikegaya

**Affiliations:** 1 Laboratory of Chemical Pharmacology Graduate School of Pharmaceutical Sciences, University of Tokyo, Tokyo 113-0033, Japan; 2 Center for Information and Neural Networks, National Institute of Information and Communications Technology, Osaka 565-0871, Japan

**Keywords:** Long-term plasticity, N-methyl-d-aspartate receptor, primary visual cortex, visual perception

## Abstract

The primary sensory neocortex generates an internal representation of the environment, and its circuit reorganization is thought to lead to a modification of sensory perception. This reorganization occurs primarily through activity-dependent plasticity and has been well documented in animals during early developmental stages. Here, we describe a new method for the noninvasive induction of long-term plasticity in the mature brain: simple transient visual stimuli (i.e., flashing lights) can be used to induce prolonged modifications in visual cortical processing and visually driven behaviors. Our previous studies have shown that, in the primary visual cortex (V1) of mice, a flashing light stimulus evokes a long-delayed response that persists for seconds. When the mice were repetitively presented with drifting grating stimuli (conditioned stimuli) during the flash stimulus–evoked delayed response period, the V1 neurons exhibited a long-lasting decrease in responsiveness to the conditioned stimuli. The flash stimulus–induced underrepresentation of the grating motion was specific to the direction of the conditioned stimuli and was associated with a decrease in the animal’s ability to detect the motion of the drifting gratings. The neurophysiological and behavioral plasticity both persisted for at least several hours and required *N*-methyl-d-aspartate receptor activation in the visual cortex. We propose that flashing light stimuli can be used as an experimental tool to investigate the visual function and plasticity of neuronal representations and perception after a critical period of neocortical plasticity.

## Significance Statement

A flashing light induces an anomalously delayed response in the primary visual cortex of mice, rats, and humans. We discovered that the delayed response, combined with conditioned stimulus, has an ability to induce long-term neuronal and behavioral plasticity in an NMDA receptor–dependent manner.

## Introduction

Cortical response properties undergo prolonged plastic changes throughout life, but plasticity capacity is known to decline with age (Frégnac et al., 1992; Frenkel et al., 2006; Levelt and Hübener, 2012). To induce *in vivo* plasticity in adult cortical networks, researchers often use invasive approaches, including electrical and iontophoretic stimulation (Frégnac et al., 1992; Schuett et al., 2001). Increasingly, however, recent studies have devised experimental protocols to induce plasticity in adult visual systems via externally presented, noninvasive visual stimuli. Although it generally takes several days to weeks for detectable changes to occur (Frenkel et al., 2006; Cooke and Bear, 2010; Kreile et al., 2011), a few rapid forms of plasticity induction, such as exposure to a photic tetanus, have also been reported (Teyler et al., 2005; Clapp et al., 2006). These plastic modifications rely largely on *N*-methyl-D-aspartate receptor (NMDAR) activity (Kleinschmidt et al., 1987; Malenka and Bear, 2004; Michmizos et al., 2011).

**Figure 1.**
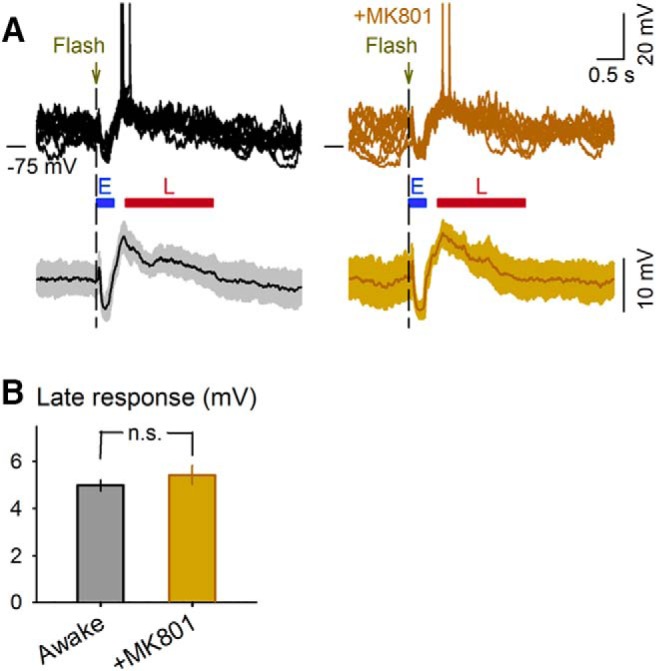
Late depolarization is independent of NMDAR activity. ***A***, *V_m_* responses of a cell to a flash stimulus before (left) and after (right) MK801 application (10 mg/kg, i.p.) in individual trials (top) and the mean ± SD of all trials (bottom, *n* = 49 and 30 trials, respectively). E and L represent early and late response periods, respectively. ***B***, Mean depolarization amplitudes of the late responses in the cell shown in ***A***. There were no significant differences between these two conditions (*P* = 0.30, *t*_77_ = 1.06, Student’s *t* test).

The primary visual cortex (V1) mediates visual perception of the external world (Leopold, 2012; Glickfeld et al., 2013; Petruno et al., 2013). V1 neurons exhibit selective responses to low-level visual features, such as color, orientation, and motion (Livingstone and Hubel, 1984; Niell and Stryker, 2008). These basic features develop during the early postnatal weeks and persist thereafter (Fagiolini et al., 1994; Rochefort et al., 2011; Hagihara et al., 2015). In adult mice, however, we report that flashing light stimuli can rapidly induce a long-term change in V1 neuronal responses. It was previously reported that a brief flashing light stimulus induces a unique V1 activity pattern that consists of a fast, transient response and a long-delayed, persistent response in both spiking activity and subthreshold membrane potentials (*V_m_*; Funayama et al., 2015; Fig. 1). Here, we found that when the delayed response is repeatedly presented with a featured visual stimulus, a long-term decrease in the V1 response is induced for an extended period of time when it is repeatedly coupled with the featured stimulus. We also confirmed a long-lasting suppressive aftereffect of flash stimuli on visual perception.

## Materials and Methods

### Ethical approval

The animal experiments were performed with the approval of the Animal Experiment Ethics Committee at the University of Tokyo (approval number: 21-6) and were performed according to the University of Tokyo guidelines for the care and use of laboratory animals.

### Animal preparation for recordings

Male C57BL/6J mice (Japan SLC) were used for the animal experiments, which included LFP and whole-cell patch clamp recordings and two-photon imaging. A single recording was obtained from each animal. The animals were housed in cages under standard laboratory conditions (12-h light/dark cycle with free access to food and water). All efforts were made to minimize the animals’ suffering and the number of animals used. Recordings were conducted on postnatal day 28 (P28) to P35 for juvenile preparations and on P68–P75 for adult preparations. All physiologic recordings were made in awake animals. Animal preparations were conducted according to the following three steps: (1) installment of a head-holding plate, (2) recovery and habituation to head fixation, and (3) craniotomy and durotomy on the day of recordings. For head plate installation, animals were anesthetized using ketamine (50 mg/kg, i.p.) and xylazine (10 mg/kg, i.p.), and 0.2% lidocaine (20 μl, i.p.) was applied under the skin on the head for analgesia. Anesthesia was confirmed based on the lack of paw withdrawal, whisker movement, and eye blink reflexes. The head skin was then removed, and the animal was implanted with a metal head-holding plate. This surgery took 30–40 min. After 2 d of recovery, head fixation training using a custom-made stereotaxic fixture was repeated for 1–3 h per day until the implanted animal learned to remain still. During and after each session, the animal was rewarded with free access to sucrose-containing water. During the final three sessions, sham experiments were conducted to habituate the animal to the experimental conditions and noise. On the last 2–3 d, the animal was maintained virtually immobile (still but awake) for >2 h. After full habituation, the animals were anesthetized using ketamine/xylazine for a craniotomy and durotomy. A craniotomy (1 × 1 mm^2^), centered 3.5 mm posterior to the bregma and 2.0 mm ventrolateral to the sagittal suture, was performed on the left cerebral hemisphere, and the dura was surgically removed. The exposed cortical surface was covered with 1.7–2.0% agar at a thickness of 0.5 mm. Throughout the experiments, a heating pad was used to maintain the rectal temperature at 37°C. Recordings were initiated after recovery from anesthesia, which was confirmed based on spontaneous whisker movements and touch-induced eye blink reflexes. Prerecording surgical treatment took 30–40 min, whereas the whole preparation, including full recovery from anesthesia, took 1–2 h. A single acute recording was conducted for each animal. The total duration of the recordings was restricted to <1 h per animal to minimize animal stress. The recorded area was confirmed via *post hoc* imaging of a fluorescent compound, sulforhodamine 101 (SR101). SR101 was dissolved to a concentration of 0.1 mm in artificial CSF (aCSF; 127 mm NaCl, 1.6 mm KCl, 1.24 mm KH_2_PO_4_, 1.3 mm MgSO_4_, 2.4 mm CaCl_2_, 26 m, NaHCO_3_ and 10 mm glucose, pH 7.3) and was pressure-injected (50 psi, 10 s) at a depth of 200 µm under the craniotomy using a glass pipette (tip diameter: 20 µm). At the end of the recording, the animals were anesthetized with urethane (1.5 mg/kg, i.p.) and killed by cervical dislocation.

### Pharmacology

d-(-)-2-Amino-5-phosphopentanoic acid (AP5) and tetrodotoxin (TTX) were dissolved in aCSF to a concentration of 300 and 10 μm, respectively, and were directly applied to the cortical surface 15 min before the recording or behavioral sessions. A cranial window was made over the V1 contralateral to the stimulated eye for physiologic experiments. Because optomotor tests and the induction of plasticity were conducted binocularly in the behavioral experiments, craniotomy was also performed bilaterally. After application of the pharmacological agents, the exposed cortices were covered with the craniotomized bone segments and mounted with dental cement before the relevant behavioral experiments. We estimated the spread of the pharmacological agents (AP5 and TTX) using SR101, which has hydrophilic properties and molecular weight similar to those drugs. The spread was limited to the visual cortical areas (data not shown). For the longevity of the pharmacological effect, previous studies have shown that superficially applied AP5 impact cortical neurons shortly after perfusion and the effect is gone in a half-day (Rema et al., 1998). The effect of superficially applied or infused TTX has been shown to last for a couple of hours and disappear gradually within 24 h (Zhuravin and Bures, 1991; Kingma et al., 1992; Lorenzini et al., 1995).

### Visual stimulation

Visual stimuli were generated using custom-designed Matlab protocols (MathWorks; RRID: SCR_001622) with Psychtoolbox extensions (Brainard, 1997; RRID: SCR_002881). A 17-inch gamma-corrected TN-LCD monitor (refresh rate: 60 Hz) was placed 30 cm from the right cornea, which was contralateral to the recording site, such that it covered 38.8° of the mouse’s horizontal visual field and 29.6° of the vertical visual field. For flash stimulation, a white screen at 240 cd/m^2^ (65 lx near the eye) was presented for 100 ms (corresponding to six video frames) at an interval of 10 s, during which a gray screen at 60 cd/m^2^ (5 lx) was continuously displayed. In all recording conditions, responses to flash stimulus during the first 25 trials were discarded because these responses might have been subject to retinal adaptation. For drifting grating stimulation, sinusoidal gratings (spatial frequency, 0.03 cpd; temporal frequency, 2 Hz; contrast, 40%, mean luminance, 60 cd/m^2^) moved in one of eight evenly spaced directions (0°, 45°, 90°, 135°, 180°, 225°, 270°, or 315°) for 1.5 s at intervals of 2–3 s for electrophysiology analyses and for 4 s at an interval of 6 s for calcium imaging. A gray screen was presented during the interval period. For each set, stimuli drifting in eight directions were presented in a pseudorandom order, and the set was repeated 10–30 times.

### Electrophysiology

The signals were amplified using a MultiClamp 700B amplifier and analyzed using pCLAMP10.1 software (Molecular Devices); the signals were then digitized at 20 kHz. The data were reduced to 2 kHz and analyzed offline using custom-designed Matlab protocols. Patch-clamp recordings were obtained from L2/3 neurons at depths of 150–350 μm from the V1 surface using borosilicate glass electrodes (3.5–6.5 MΩ) that were pulled using a P-97 puller (Sutter Instruments). The electrode tips were lowered perpendicularly into the V1 using a DMX-11 electric manipulator (Narishige). For cell-attached recordings, pipettes were filled with aCSF. For whole-cell recordings, the intrapipette solution consisted of the following (in mm): 130 K-gluconate, 10 KCl, 10 Hepes, 10 Na_2_-phosphocreatine, 4 Mg-ATP, 0.3 Na_2_GTP, and 0.2% biocytin, all adjusted to pH 7.3. For voltage-clamp recordings, the intrapipette solution consisted of the following (in mm): 130 CsMeSO_4_, 4 tetraethylammonium-Cl, 10 Hepes, 10 Na_2_-phosphocreatine, 0.5 EGTA, 4 MgATP, 0.3 Na_2_GTP, 2 QX-314, and 0.2% biocytin, all adjusted to pH 7.3. Because the ionic composition of extracellular fluid in the brain is similar to that of aCSF, excitatory and inhibitory postsynaptic currents (EPSCs and IPSCs, respectively) were dominant at clamped voltages of –74 and 0 mV, respectively (Abe et al., 2014). Based on the assumptions that leak currents were blocked by intracellular cesium ions and that the equilibrium potentials for EPSCs and IPSCs were 0 and –74 mV, the excitatory and inhibitory conductance at any given point in time was estimated as *G_e_* = EPSC/(–74) and *G_i_* = IPSC/74. Experiments in which the series resistance exceeded 70 MΩ or changed by >15% during the recording session were discarded. For each neuron, the spike responses to a brief inward current were examined, and regular spiking neurons were selected as putative pyramidal cells for the subsequent analyses. As shown in Figs. 3*C*, 3*D*, and 5, we analyzed the data from neurons that fired at >0.2 Hz in response to visual stimulation to ensure the assessment of their direction selectivity. LFPs were recorded at a depth of 250 μm from the V1 surface, which approximately corresponds to the lower border of L2, using borosilicate glass pipettes (1–2 MΩ) filled with aCSF. The traces were bandpass filtered between 1 and 250 Hz. Visually evoked LFPs contain stimulus-locked, fast transient deflection on top of local oscillatory components. The stimulus-locked components were obtained by averaging the traces across all trials and were subtracted from the single trial traces to separately analyze the oscillatory components. The oscillatory power was measured using Morlet wavelet decomposition on 42 scales between 1 and 90 Hz. After applying the wavelet transformation, the values for the first and last 2 s were removed to reduce the edge effect. Stimulus-evoked power changes from prestimulus baseline activity for each time point and frequency were calculated using the following equation (Ray and Maunsell, 2010):

(1) $$P\left(t,\omega \right)=10\times \left[{\text{log}}_{\text{1}0}W\left(t,\omega \right)-{\text{log}}_{\text{1}0}W_{B}\left(\omega \right)\right],$$

where *W*(*t*,ω) is the mean wavelet power averaged across trials at time *t* and frequency ω, and *W_B_*(ω) is the baseline wavelet power calculated by averaging the prestimulus *W*(*t*,ω) across time:

(2) $$W_{B}\left(\omega \right)=1/\text{T}\omega^{t=t_{0}∼t_{0}+\text{T}}W\left(t,\omega \right),$$

where *t*_0_ = –3000 ms and *T* = 2000 ms. The power spectra were generated by averaging *P*(*t*,ω) within the corresponding time period at a given frequency. Time–power plots were generated by averaging *P*(*t*,ω) across the relevant frequencies for each time point.

### Two-photon calcium imaging

Each mouse was mounted on a stereotaxic frame placed on the stage of an upright microscope (BX61WI; Olympus). Cortical neurons were loaded with Fura-2 AM, a calcium-sensitive fluorescent dye, under online visual guidance via two-photon laser scanning (FV1000; Olympus). Fura-2 AM was dissolved to a concentration of 10 mm in DMSO containing 10% pluronic acid and diluted to a final concentration of 1 mm in aCSF containing 0.1 mm SR101. This solution was pressure-injected (50–100 mbar for 10 s) into the V1 at a depth of 150–250 µm from the surface using a glass pipette (tip diameter: 10–30 µm). The pipette was carefully withdrawn, and the craniotomized area was sealed with 2% agar and a glass coverslip. After 50–70 min, which allowed time for dye loading into the neuronal soma and washout of extracellular dyes, two-photon images of the Fura-2 fluorescence from V1 L2/3 neurons were obtained. Neurons and astrocytes were discriminated based on astrocyte-specific staining with SR101 (Nimmerjahn et al., 2004). Fura-2 and SR101 were excited using a mode-locked Ti:sapphire laser at wavelengths of 800 and 910 nm, respectively (100-fs pulse width, 80-MHz pulse frequency; Maitai HP; Spectra Physics; Sohya et al., 2007). Fluorescent light was collected using a water-immersion objective lens (20×, numerical aperture 0.95; Olympus). Videos were recorded from a 512 × 512-μm area (512 × 512 pixels) at 2 frames/s using FV10-ASW software (version 3.0; Olympus, RRID:SCR_014215). The fluorescence change relative to baseline (Δ*F*/*F*) was calculated for each recorded neuron. The Δ*F*/*F* was averaged over the 2-s stimulus period and over the trials and was defined as the response amplitude of the neuron. Neurons were selected for analysis as follows. First, neurons in the imaging plane that exhibited significant visual responses above the baseline (*P* < 0.05, paired *t* test) in both the pre- and poststimulus pairing recording sessions were chosen. Among these, the cells with a significant orientation selectivity index (OSI) in the prepairing sessions were selected for analysis. The OSI was defined according to the following equation:

(3) $$\text{OSI}=\frac{\sqrt{{\left(\sum^{\;}R_{\theta }\sin2\theta \right)}^{2}+{\left(\sum^{\;}R_{\theta }\cos2\theta \right)}^{2}}}{\sum^{\;}R_{\theta }},$$

where *R*_θ_ is the mean response amplitude to a grating with direction θ (Swindale, 1998). For each cell, the OSI was compared with its chance level, which was estimated using a conventional random resampling method in which 1000 surrogates were generated by randomly shuffling all trials regardless of θ.

### Normalized direction tuning

The direction tuning curve was calculated by normalizing the grating-induced neuronal responses (calcium signals and spikes) for each stimulus direction according to tuning(θ) = *R*(θ)/Ʃ *R*(θ) × 100 (θ = 0°, 45°, 90°, 135°, 180°, 225°, 270°, and 315°), where *R*(θ) indicates (a) the mean amplitude of the calcium signal (|*ΔF/F*|) or (b) the sum of the spike counts in all trials for a given stimulus with a moving direction θ. For calcium signals, *R*(θ) could have a negative value because the level of spontaneous activity is subtracted from the level of the stimulus-induced activity. In this case, we adjusted the minimal *R*(θ) of the cell to zero by uniformly raising *R*(θ) levels. Under ideal conditions, the mean tuning value for each direction is expected to be 12.5% (100%/8 directions, varied among cells). Changes in the direction tuning curves (Δtuning) were calculated as percentage points.

### Modulation index

To estimate the change in response by a preceding flash in the pairing session, we calculated (*R_p_* – *R_b_*)/(*R_p_* + *R_b_*) as the modulation index. *R_p_* and *R_b_* represent the mean responses (calcium signal amplitudes or firing rates) to the grating drifting in the paired direction during the in-pairing period and the prepairing baseline period, respectively.

### Excitation index

To quantify the excitation-to-inhibition balance during flash-evoked late responses, we defined $\left(\bar{\Delta G_{e}}-\bar{\Delta G_{\iota }}\right)/\left(\bar{\Delta G_{e}}+\bar{\Delta G_{\iota }}\right)$ as the excitation index. $\bar{\Delta G_{e}}$ and $\bar{\Delta G_{\iota }}$ represent the man excitatory and inhibitory conductances during the entire late-response period after subtraction of prestimulus baseline. This value ranges between –1 and 1 when both $\bar{\Delta G_{e}}$ and $\bar{\Delta G_{\iota }}$ are higher than zero, which is mostly the case (i.e., a flash increases both excitatory and inhibitory conductances). However, in one of six recorded neurons, $\bar{\Delta G_{\iota }}$ was slightly but not significantly less than zero. In this neuron, the excitation index was plotted as 1; note that the true value was 1.03.

### Plasticity induction

Visual stimuli were presented monocularly for the physiologic experiments and binocularly in a display configuration for the behavioral experiments. A sinusoidal drifting grating [spatial frequency, 0.03 and 0.17 cycles per degree of visual angle (cpd) for physiology and behavior, respectively; temporal frequency, 2 Hz; contrast, 40%] was presented for 1.5 s in one of four movement directions (0°, 90°, 180°, and 270°) at the same probability. The drifting grating stimuli were applied at pseudorandom intervals of 1–2 s, during which a gray screen was continuously displayed. In Fig. 3*A*, the durations of the drifting gratings and the interstimulus intervals were prolonged to 2 and 5.5 s, respectively, to reliably evaluate the evoked calcium responses during stimulus pairing. To induce plasticity, a 100-ms white-screen flash at a luminance of 240 cd/m^2^ (65 lx) was presented 400 ms before a grating drifted in one specific direction (paired stimulus), whereas no flashes were applied before the gratings drifted in the other directions. In Fig. 5, a depolarizing or hyperpolarizing current was injected into a patch-clamped neuron while a grating drifted in one specific direction (paired stimulus). The amplitude of the injected current was set to generate a *V_m_* change of ±10 mV. In each set, the directions of the drifting gratings were pseudorandomly ordered, and the set was repeated 30–50 times, which corresponded to 30–50 and 90–150 presentations of paired and nonpaired stimuli, respectively, and required ∼7–10 min.

### Virtual optomotor system

The apparatus was located in a dark, soundproofed room. Room temperature was maintained at 25°C during the experiment. A virtual cylinder comprising a vertical sinusoidal grating (0.17 cpd, 40% contrast) was displayed in the 3D coordinate space on four 24-inch monitors (refresh rate: 60 Hz) that were arranged in a quadrangular arena. The images on the monitors were extended using two mirrors on the top and bottom of the arena. A platform (a white acrylic disk, ϕ = 6.0 cm) was positioned 13.5 cm above the bottom mirror. During each experiment, a single male P68–P75 C57BL/6J mouse was placed on the platform and allowed to move freely. The behavior of the mouse was monitored using a camera (Logicool HD Webcam C615; Logitech) that was attached above a small hole in the top mirror. Vertical gratings that drifted leftward or rightward (temporal frequency: 0.5 Hz) were presented simultaneously on all four screens for 2 s with a random interval between 2 and 4 s. From the animal’s point of view, the virtual cylinder appeared to rotate around the platform at an angular velocity of 5°/s. Mice typically tracked the grating with reflexive head movements in concert with the rotation direction. The drifting directions were randomly alternated, and the rotations were repeated 120 times in one session, each of which required ∼10 min. The animals were habituated to the system before the first behavioral test by experiencing at least one full session. When the mice slipped or jumped down from the platform during the test, they were manually returned to the platform, and the test was resumed. If the animal’s head appeared to track a cylindrical rotation, the trial was counted as a success. Manual counting was validated by an independent trained researcher who was blinded to the experimental conditions. The experimenter was also blinded to the treatment. The trials in which a mouse was grooming or moving extensively were excluded from the analyses (invalid trials). The success rate was calculated as the ratio of successful trials to the total number of valid trials. We determined that the tracking rate was reduced by local injection of tetrodotoxin into the V1. This finding appears to be inconsistent with a previous report that showed that V1 lesions induced no effect on optomotor behavior (Douglas et al., 2005); however, in the previous study, the V1 was chronically ablated via tissue aspiration. Our acute inactivation was milder and reversible and, thus, is more suitable for the examination of the specific involvement of the V1.

### Statistics

**Figure 2.**
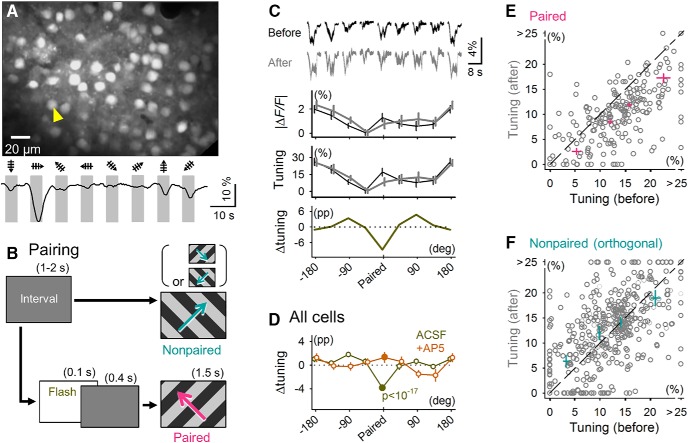
Flash conditioning induces long-term suppression of visually evoked calcium responses in V1 neurons. ***A***, Two-photon image of Fura-2–labeled L2/3 neurons. The bottom trace indicates a raw fluorescence change (|Δ*F*/*F*|) in the neuron marked by the yellow arrowhead in response to gratings drifting in eight directions of motion. ***B***, Diagram of the stimulus pairing protocol. Flashes were applied 0.4 s before showing the gratings drifting in a specific direction (paired). This flash-to-drifting grating stimulus pairing was repeated 30 times within a period of 420 s. During stimulus pairing, no flashes were applied preceding gratings that drifted in other directions (nonpaired). ***C***, Stimulus pairing-induced changes in the calcium response of a representative cell. From top to bottom: means ± SEMs (shown as the area) of visually evoked fluorescence traces for all eight directions, mean ± SEM response amplitudes, normalized tuning curves ± SEM, 5–10 min before and 5–10 min after stimulus pairing, and tuning curve change (Δtuning; pp, percentage point). The top traces are ordered according to the labeling of the *x*-axis of the bottom plot. ***D***, Δtuning was calculated as means ± SEMs of all imaged cells from mice with (+AP5, *n* = 113 neurons from 4 mice) or without (aCSF, *n* = 214 neurons from 10 mice) local application of AP5 to the V1. ***E***, Distribution of raw tuning values for the paired stimuli versus the initial tuning value. Each circle indicates a single cell. Cross symbols represent means ± SEMs for data points in quartile sections. The line is the diagonal. ***F***, Same as in ***E*** but for the drifting gratings orthogonal to the paired stimuli.

**Figure 3.**
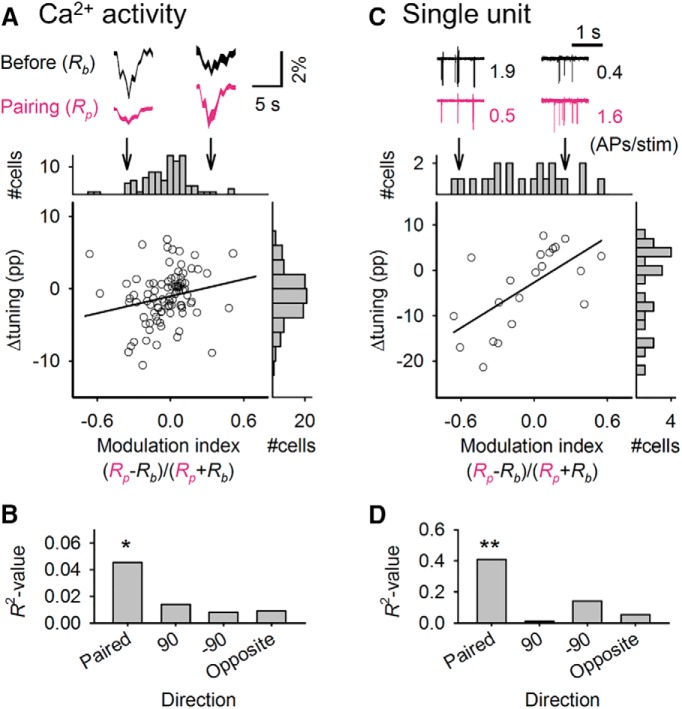
Flash stimulus–induced plastic changes in visual responsiveness are correlated with flash stimulus–induced activity alterations during stimulus pairing. ***A***, Δtunings for the paired stimulus of individual cells are plotted against the response modulation during stimulus pairing. Each axis includes a collapsed histogram. The black line represents the linear regression. The top traces represent means ± SEMs (shown as the area) of visually evoked calcium responses to gratings drifting in the paired direction before (black) and during (red) stimulus pairing for two representative cells. ***B***, Coefficients of determination (*R* ^2^) between the modulation index and Δtuning for the four directions used in the flash stimulus pairing were determined using simple linear regression. *P = 0.044, *r*_90_ = 0.21. ***C***, ***D***, Data from single-unit conditioning experiments are shown in the same manner as in ***A*** and ***B***. Numbers in close proximity to the unit traces indicate the average action potential (AP) rates per trial in response to the gratings drifting in the paired direction. **P = 1.8 × 10^–3^, *r*_21_ = 0.64.

**Figure 4.**
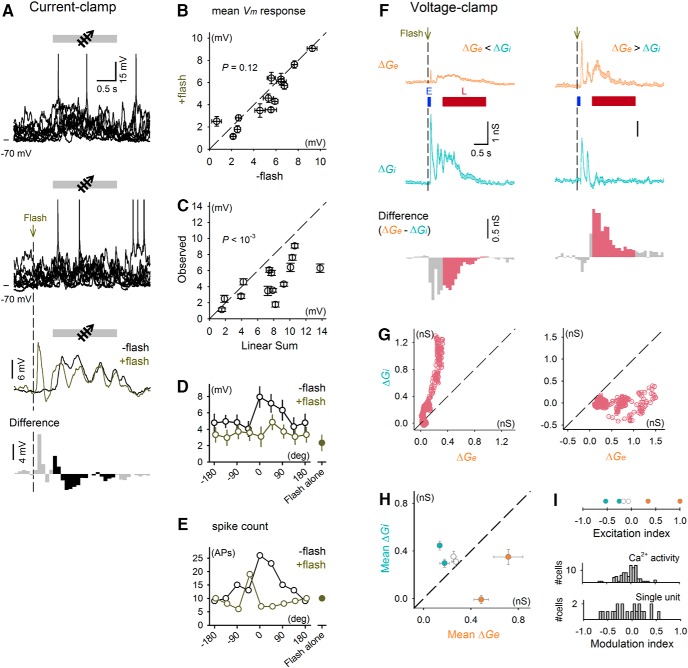
Flash stimulus–evoked excitatory and inhibitory synaptic inputs underlie sublinear summation of flash and drifting grating stimuli. ***A***, *V_m_* responses of a current-clamped cell to a drifting grating with and without a preceding flash stimulus. From top to bottom: representative traces of individual trials for a drifting grating alone or for a drifting grating with a preceding flash stimulus, mean responses to these two types of stimuli, and differences at a bin size of 100 ms. ***B***, Means ± SEMs of *V_m_* responses to drifting gratings averaged over all directions of 13 recorded neurons. Trials with preceding flashes were plotted against trials with drifting gratings alone. *P* = 0.115, *n* = 14, *t*_13_ = 1.01. Paired *t* test. ***C***, Means ± SEMs of *V_m_* responses to drifting gratings preceded by flashes over all directions were plotted against the linear sum of the mean *V_m_* responses to drifting gratings alone and flash-evoked late responses. *P* = 9.7 × 10^–4^, *t*_13_ = 4.20. ***D***, ***E***, Tuning curves of the mean *V_m_* responses (***D***) and total number of spikes across all trials (***E***) for the example cell shown in ***A***. Error bars represent SEMs. ***F***, Flash stimulus–evoked changes in excitatory (orange, Δ*G_e_*) and inhibitory (cyan, Δ*G_i_*) synaptic conductances of two representative L2/3 neurons. The lower histograms indicate the differences between Δ*G_e_* and Δ*G_i_* using a bin size of 100 ms. ***G***, Δ*G_e_* and Δ*G_i_* during the flash stimulus–evoked late responses plotted for every 5-ms bin. The data were obtained from the same neurons shown in ***F***. ***H***, Mean Δ*G_e_* and Δ*G_i_* of the late responses of all six recorded neurons. Error bars represent SEMs of trial-by-trial variability of conductances during the late response period. Cells that significantly showed Δ*G_e_* or Δ*G_i_* dominance are plotted in orange or cyan, respectively (*P* < 0.05, Student’s *t* test). (I) Top, excitation index calculated from the mean Δ*G_e_* and Δ*G_i_* during the late responses of the cells shown in ***H***. Bottom, modulation index in Fig. 3*A* and *C* were replotted for comparison.

**Figure 5.**
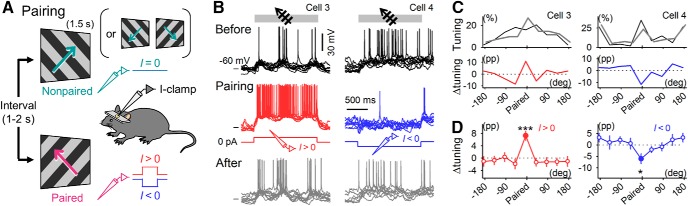
Visually evoked responses are bidirectionally modifiable by current injection. ***A***, Diagram of the stimulus pairing protocol. Direct currents were injected for 1.5 s to depolarize (*I* > 0) or hyperpolarize (*I* < 0) patch-clamped neurons during 1.5-s gratings drifting in a specific motion direction. The coupling of current injection with the grating stimulus was repeated 30 times. During stimulus pairing, no current was injected for gratings drifting in other directions. ***B***, Representative *V_m_* traces 5 min before (top), during (middle), and 5 min after (bottom) stimulus pairing with depolarizing (cell 3) or hyperpolarizing (cell 4) currents. Ten consecutive sweeps are shown per condition. ***C***, Normalized tuning curves for the grating-evoked spike counts before (black) and after (gray) stimulus pairing (top) and differences (Δtuning, bottom). ***D***, Δtunings of 16 depolarized (left) and 11 hyperpolarized (right) cells are calculated as means ± SEMs. ***P = 2.6 × 10^–4^, *t*_15_ = 4.7; *P = 0.019, *t*_10_ = 2.8; paired *t* test.

Statistical relevance was evaluated with paired *t* tests in Figs. 2–5 and 7 after confirming normality using *F* test, Student’s *t* tests for Fig. 1, and simple linear regression tests for Fig. 3. For Fig. 2, the general effect of the flash stimulus paring on tuning curve was also addressed by one-way ANOVA. The *P* values are indicated in the figures as follows: n.s. (not significant), ^§^*P* < 0.05, ^*^P < 0.05, ^**^P < 0.01, and ^***^P < 0.001.

## Results

### Plasticity of visual neuronal responses to flash-paired stimuli

We previously showed that a flashing light stimulus induces prolonged offline depolarization in V1 neurons (Funayama et al., 2015, 2016; Fig. 1). This late depolarization (L in Fig. 1, in comparison with early depolarization, E) raises the *V_m_* closer to the spike threshold and, therefore, may lead to spike responses when an otherwise subthreshold visual stimulus arrives during the late response period (pairing). Thus, we expected that repeated pairings would induce a long-term potentiation (LTP)-like facilitation in response to visual stimuli because synapses that induce suprathreshold activity are predicted to be potentiated according to the activity-dependent rule of synaptic plasticity; that is, the synaptic connection between two neurons undergoes LTP when they fire together and long-term depression (LTD) when they fire asynchronously (Hebb, 1949; Stent, 1973; Tamura et al., 1992; Zucker, 1999).

To assess the plasticity-inducing effect of a flash stimulus, we performed functional multineuron calcium imaging of V1 neurons from awake juvenile (P28–P35) mice. Thus, visual responses of L2/3 neurons to drifting grating stimuli were monitored (Fig. 2*A*). V1 neurons are known to exhibit selective responses to low-level visual features, such as color, orientation, and motion, and their direction selectivity is one of the most well-documented characteristics (Livingstone and Hubel, 1984; Niell and Stryker, 2008; Herzog and Clarke, 2014). Based on the |Δ*F/F*| amplitudes of calcium transients, we calculated the normalized direction tuning curve (hereafter, referred to simply as tuning; see Methods for more detail).

To induce plasticity, we paired a drifting grating in a specific direction (paired direction) 0.4 s after a flash, which corresponds to the onset of the flash-evoked late response (Fig. 2*B*), and we repeated this flash-to-grating pairing 30 times at random intervals. During this period, gratings in other directions (unpaired directions) were presented intermittently in a pseudorandom order without preceding flashes (see Methods). The paired direction was randomly selected without considering the direction preferences of individual neurons. After 5 min, we again measured the calcium responses to drifting gratings and calculated the difference in the direction tuning (Δtuning) between the pre- and postpairing sessions (Fig. 2*C*). As a consequence of flash stimulus pairing, the shapes of direction tuning curves were significantly changed (Fig. 2*D*, *P* = 3.7 × 10^–18^, *F*_7_ = 14.3, one-way ANOVA; *n* = 214 cells from 10 mice). In contrast to our expectations, the calcium responses to grating drifting in the paired directions were, on average, selectively suppressed (*P* = 1.1 × 10^–18^, *t*_213_ = 9.7, paired *t* test). This LTD-like change in the visual responses occurred regardless of the initial tuning level to the paired stimulus (Fig. 2*E*). This plasticity did not occur in mice that received 300 μM AP5, an NMDAR antagonist, via local perfusion into the V1 15 min before pairing (Fig. 2*D*; *P* = 0.18, *t*_112_ = 1.3, paired *t* test; *n* = 113 cells from four mice). These results suggest that flash-induced LTD-like plasticity depends on the activation of NMDAR within the early visual cortices. A blockade of NMDARs, which have relatively slow kinetics and therefore are known to be involved in persistent neuronal activity (Wang, 1999; Wang et al., 2013), by intraperitoneal MK801 administration did not *per se* affect the late depolarization evoked by a flash alone (Fig. 1*A*, *B*).

To address the apparent inconsistency between our prediction and the results, we focused on neuronal responses during flash stimulus pairing, which we predicted to be higher than in the prepairing period because of flash-evoked late depolarization. We monitored calcium activity in 90 neurons from two mice and single-unit recordings in 21 neurons from 19 mice before, during, and after pairing (Fig. 3). Then, we compared Δtuning to the flash-induced changes in the response during pairing, which was calculated as the modulation index (see Methods). This index ranged from –1 to 1 and displayed higher values when a flash stimulus increased the response to the following paired stimulus compared with the response to drifting gratings alone. There was a large variability in the modulation index (–0.03 ± 0.19 calcium activity and 0.02 ± 0.34 single-unit, mean ± SD), indicating that the flash-induced modulations of the responses to drifting gratings varied among neurons and that a substantial proportion of neurons exhibited negative values for the modulation index, in contrast to our expectation that flashes would enhance neuronal responses to subsequent stimuli. Similar variability in the flash-induced modulation was confirmed by whole-cell current-clamp (*I* = 0) recordings of V1 L2/3 neurons (Fig. 4*A*). The effect of preceding flashes on the *V_m_* responses to drifting gratings varied among neurons (Fig. 4*B*). The existence of negative modulation by flashes in many neurons suggests that the flashing stimulus increased the net membrane conductance and reduced the responsiveness to external stimuli. To examine this possibility, we voltage-clamped V1 L2/3 neurons and determined that flash-induced late depolarization was composed of persistent increases in both excitatory and inhibitory conductances (*G_e_* and *G_i_*, respectively; Fig. 4*F*, *G*). In four of six neurons recorded, stimulus-induced increases in *G_e_* (Δ*G_e_*) during the late response period were significantly different from those of Δ*G_i_* (Fig. 4*H*), which indicates that excitatory and inhibitory synaptic barrages were not balanced during the late responses. We calculated the excitation index to estimate the variability of the *G_e_*-to-*G_i_* ratio during the late responses (see Methods). As with the modulation index, the excitation index also varied from neuron to neuron (Fig. 4*I*; 0.06 ± 0.54) and may underlie the variation in the modulation index. Importantly, Δtuning of the paired stimulus correlated weakly but significantly with the modulation index (Fig. 3, *P* = 0.044 and 1.8 × 10^–3^, *r^2^* = 0.05 and 0.41 for calcium activity and single-unit conditioning, respectively). These correlations were specific to the paired direction (Fig. 3*B*, *D*). In other words, cells in which the flash stimuli evoked an increase in the response to the paired grating during pairing subsequently exhibited LTP-like facilitation of the responses, whereas cells in which flash stimuli evoked a decrease in this response during pairing exhibited LTD-like suppression. This correlation resembles the bidirectional modifications predicted by the activity-dependent rule of synaptic plasticity (Bear and Abraham, 1996). However, the mean values of the modulation index were nearly zero, indicating that the sign of long-term plasticity was determined not simply by the sign of an instantaneous effect (see Discussion).

To further confirm the activity-dependent plasticity in awake mice, we replaced flash stimulation with direct current injection using the current-clamp technique. The recorded neurons were depolarized or hyperpolarized by ∼10 mV while drifting gratings in a specific direction were presented (paired stimulation; Fig. 5*A*). During grating stimulation, robust spike trains were generated by depolarization, whereas few spikes occurred during hyperpolarization (Fig. 5*B*). After stimulus pairing with depolarization, the responses to the paired stimuli were enhanced (Fig. 5*C*, *D*, *P* = 2.6 × 10^–4^, *t*_15_ = 4.7, paired *t* test; *n* = 16 cells). On the other hand, stimulus pairing with hyperpolarization resulted in depression of the responses to paired stimuli (*P* = 0.019, *t*_10_ = 2.8; *n* = 11 cells).

### Plasticity of behavioral responses to visual stimuli paired with a flash stimulus

**Figure 6.**
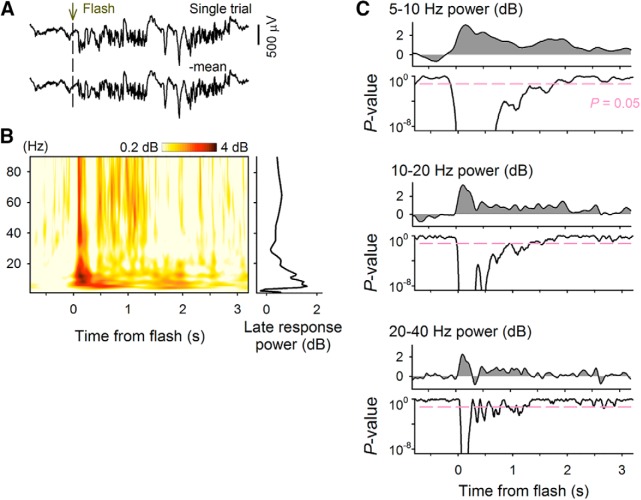
Late LFP responses are evoked by flashing lights in adult mice. ***A***, Raw (top) and mean-subtracted (bottom) LFP traces recorded from the V1 of an adult mouse. ***B***, Pseudocolored time–frequency map (left) of the mean-subtracted LFP trace and the power spectra of the late response (right). ***C***, Time change in the LFP power and *P* value from baseline activity at various frequencies.

We also evaluated the behavioral consequences of flash stimulus–induced plasticity on neuronal responses using P68–P75 mice, which are more broadly regarded as adult in the field of visual plasticity (Fagiolini and Hensch, 2000; Sawtell et al., 2003). As in a previous study with juvenile mice (Funayama et al., 2016), we confirmed late LFP responses induced by flashing lights in these mature animals; that is, the broadband power of LFPs increased and persisted for seconds after flashes (Fig. 6).

**Figure 7.**
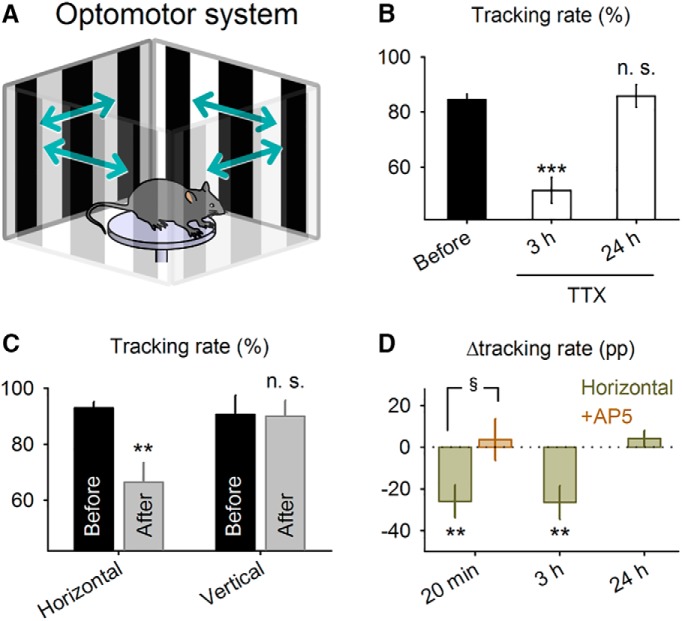
Flash conditioning induces prolonged attenuation of visual perception in adult mice. ***A***, An adult (P68–P75) mouse was placed in the arena surrounded by four screens that intermittently displayed gratings drifting leftward or rightward, and the probability that the mouse moved its head in the same direction as the drifting gratings (tracking rate) was measured. ***B***, Tracking rates were measured 24 h before and 3 and 24 h after local application of tetrodotoxin to the V1. ***P = 1.2 × 10^–4^ vs. before, *t*_8_ = 6.9; paired *t* test. ***C***, Tracking rates were compared 1 d before and 20 min after flash pairing with horizontally or vertically drifting gratings. **P = 7.1 × 10^–3^, *t*_11_ = 3.3; paired *t* test. ***D***, Changes in the tracking rates 20 min, 3 h, and 24 h after pairing with horizontally drifting gratings. AP5 was locally injected into the V1 15 min before stimulus pairing (+AP5). 20 min: **P =7.1 × 10^–3^, *t*_11_ = 3.3; 3 h: **P = 7.7 × 10^–3^, *t*_10_ = 3.3; paired *t* test; ^§^*P* = 0.029, *t*_22_ = 2.3, Student’s *t* test.

Vision-based reflex in mature mice was assessed using a virtual optomotor test, a psychophysical assay that does not require operant training (Prusky et al., 2004). Animals were placed on a circular platform that was surrounded by four computer displays. They were then presented with gratings that drifted either rightward or leftward on the displays for 2 s (Fig. 7*A*). We calculated the probability (i.e., the tracking rate) that the mice moved their heads toward the same direction of the drifting gratings. The average tracking rate was 84.5 ± 5.9% (mean ± SD of nine mice, 15–41 trials per mouse), which was reduced by a local injection of tetrodotoxin into the V1 (Fig. 7*B*, before vs. 3 h after: *P* = 1.2 × 10^–4^, *t*_8_ = 6.9, *n* = 9 mice; before vs. 1 d after: *P* = 0.82, *t*_8_ = 0.24, paired *t* test, *n* = 9 mice). Thus, the tracking behavior depended, at least in part, on V1 neuronal activity, although the optomotor system has been primarily used as a tool to estimate subcortical visual function (Wang et al., 2009; see Discussion).

We repeated the optomotor test before and after pairing a horizontally or vertically drifting grating with a preceding flash stimulus. The same protocol and time course that were used in Fig. 2*B* were adopted, except that the stimuli were presented binocularly during the pairing, and the spatial frequency was the same as in the optomotor tests (see Methods). After stimulus pairing, the tracking rate for the paired direction was significantly decreased (Fig. 7*C*, horizontal: *P* = 7.1 × 10^–3^, *t*_11_ = 3.3, *n* = 12 mice; vertical: *P* = 0.82, *t*_5_ = 0.24, paired *t* test, *n* = 6 mice). This effect was detected both 20 min and 3 h after stimulus pairing, and the tracking rate recovered to the baseline level by 24 h after stimulus pairing (Fig. 7*D*, before vs. 20 min after: *P* = 7.1 × 10^–3^, *t*_11_ = 3.3, *n* = 12 mice; before vs. 3 h after: *P* = 7.7 × 10^–3^, *t*_10_ = 3.3, *n* = 11 mice; and before vs. 1 d after: *P* = 0.30, *t*_8_ = 1.1, *n* = 9 mice, paired *t* test). As in the case of LTD-like suppression of neuronal activity, the behavioral effect of flash stimulus pairing was dependent on NMDARs; local AP5 application to V1 at 15 min before stimulus pairing prevented the flash stimulus–induced reduction in the tracking rate (Fig. 7*D*, 20 min vs. AP5: *P* = 0.029, *t*_22_ = 2.3, *n* = 12 mice each, Student’s *t* test).

## Discussion

We discovered that the flashing light stimuli induced LTD-like attenuations in neuronal and behavioral responses to visual stimuli that had been paired with flashing stimuli, although we do not present a direct causal effect for the flash-induced late responses. This flash stimulus–induced plasticity was prevented by local injection of an NMDAR antagonist into the V1. Therefore, this plasticity occurred within the V1 network, which includes local intra-V1 connections and cortical afferents to the V1. Our findings do not necessarily exclude the possibility that plasticity also occurred in extra-V1 areas, such as anterior parts of the neocortex and the superior colliculus (Liang et al., 2015).

Although the optomotor test we used here has previously been associated with subcortical structures, a study has suggested that cortical ablation leads to a slight reduction in the score shortly after a treatment (on the next day of cortical aspiration; Douglas et al., 2005). By virtue of a relatively immediate test after V1 ablation (3 h after TTX application), the present study confirmed that optomotor behaviors rely largely on V1 cortical activity. This result, together with previous studies, seems to show that subcortical compensation appears to develop gradually after V1 ablation.

The V1 network is intertwined in a complex manner, and the V1 dendritic trees within a neuron show variously tuned hotspots, which presumably reflect local synaptic activity, regardless of the preferred direction of the spike output of the neuron (Jia et al., 2010). Theoretically, the spike preference can even be shaped by mixed synaptic inputs (Hansel and van Vreeswijk, 2012), and recent experimental data support this view (Chen et al., 2013; Wilson et al., 2016). Thus, even without large-scale V1 network rewiring, a small change in the V1 synaptic weights alone may be enough to modify the preferential response of a cell. This mechanism may underlie the rapid induction of V1 plasticity observed in this study. In this study, neuronal plasticity was observed in juvenile (P28–P35) animals and was confirmed behaviorally in adult (P68–P75) animals. Therefore, the developmentally available period for the induction of plasticity seems to have a wide range and needs to be clarified in future studies.

Because V1 is known to have selective responsiveness to other visual features such as color (Livingstone and Hubel, 1984), it is interesting to examine whether the effect of a flashing light is also found for those other features. Recent studies have examined how V1 neurons represent natural images (Baudot et al., 2013; Froudarakis et al., 2014); therefore, in future studies, a more generalized consequence of repetitive exposures to flashing lights should be addressed with regard to natural viewing situations.

Flash stimulus–induced V1 plasticity appeared to be activity dependent in that the long-term consequences of stimulus pairing were consistent with an instantaneous change in the response amplitude evoked by a preceding flash during the pairing period. By manipulating the *V_m_* of a single neuron by the current-clamp technique, we replicated the stimulus-selective and activity-dependent bidirectional plasticity of the visual representation. However, whereas the overall response change after the flash stimulus pairing was depression, the mean instantaneous changes calculated as the modulation index were nearly zero. This result suggests that the sign of long-term plasticity was determined not simply by the sign of an instantaneous effect but also by the relative significance of the paired stimulus; the response to weak (drifting grating) stimuli might be obscured by a strong (flash) stimulus, thereby undergoing LTD. If a combination of a flash stimulus and a drifting grating evokes the same level of neuronal activity as a drifting grating alone, a relative contribution of the grating stimulus to the evoked activity would be smaller because of the presence of a flash late response. In other words, flash late responses might have masked neuronal activity evoked by the ensuing drifting grating and thereby shifted the activity-dependent function toward LTD. We do not exclude the potential contributions of other factors, in addition to the postsynaptic activity level. Both *in vivo* and *in vitro* studies have indicated that neuromodulators, including acetylcholine, dopamine, and noradrenaline, play permissive roles in plasticity induction (Bröcher et al., 1992; Bao et al., 2001; Wespatat et al., 2004). These modulators are related to arousal or vigilance and are released when an animal is exposed to an alarming or rewarding stimulus (Horvitz, 2000).

Repeated visual training is known to improve visual task performance, and several studies of humans and nonhuman primates have shown that V1 activity changes after perceptual learning (Crist et al., 2001; Neary et al., 2005; Li et al., 2008). In the mature brain, visual plasticity can also be induced via passive exposure to behaviorally irrelevant stimuli. One well-known protocol is to repeatedly present a particular stimulus that causes stimulus-selective potentiation of a neuronal response in mice (Frenkel et al., 2006) and of a signal detection power in humans (Watanabe et al., 2002; Seitz et al., 2009). In particular, Cooke et al. (2015) found that repeated presentation of visual stimulation results in a characteristic-specific reduction of a visually driven motor response in an NMDAR-dependent manner. In adult animals, although full potentiation generally requires a few sessions (or days), the effect may become visible within 2 min after stimulus presentation at a high repetition frequency (photic tetanus; Teyler et al., 2005; Clapp et al., 2006). Presentation of two visual stimuli with a short time lag (<50 ms) is also known to induce visual plasticity within ∼2 min, but the plasticity persists for only <30 min (Yao and Dan, 2001; Fu et al., 2002). The lack of visual input after monocular or binocular deprivation also functions as a trigger of visual plasticity or metaplasticity (Sawtell et al., 2003; He et al., 2006), but this form of plasticity requires 5–10 d in the adult brain. Among this rich literature, the present study provides a novel form of rapid and long-lasting plasticity in the mature visual system. Unlike stimulus-selective potentiation by repetitive presentation, flash stimuli that were used in combination with subsequent modest visual stimuli resulted in a texture-selective decrease in the visual detection ability. Although the intensity of the flashing stimulus employed here (Δluminance = ∼60 lx) is comparable to that experienced in our daily lives, such as that of a camera strobe, we rarely encounter such repeated, specific exposures to visual stimuli. However, our findings suggest the potential utility of using flashing light stimuli to investigate neuronal and behavioral plasticity after the critical period of neocortical plasticity and visual function.

## Synthesis

Reviewing Editor: Leonard Maler, University of Ottawa Decisions are customarily a result of the Reviewing Editor and the peer reviewers coming together and discussing their recommendations until a consensus is reached. When revisions are invited, a fact-based synthesis statement explaining their decision and outlining what is needed to prepare a revision will be listed below. The following reviewer(s) agreed to reveal their identity: Christian Casanova.

Both reviewers agree that the Ms is now substantially improved and suitable for publication in eNeuro.

One reviewer suggests two minor changes that do not require re-review

In page 16, line 339, it would be appropriate to describe quantitatively the range of activation of the excitatory and inhibitory conductances. In the same paragraph, the authors suggest that this Ge-to-Gi variation could underlie the variation in the modulation index. Is there a way to demonstrate this relation statistically?

The authors should inform the editor of their response to these comments when they submit the final version of the Ms.
